# Climate action has valuable health benefits

**DOI:** 10.1097/EE9.0000000000000288

**Published:** 2024-01-12

**Authors:** Aina Roca-Barceló, Mary B. Rice, Yanelli Nunez, George Thurston, Gudrun Weinmayr, Kurt Straif, Charlotte Roscoe, Kristie L. Ebi, Zorana Jovanovic Andersen, Audrey de Nazelle, Maya Negev

**Affiliations:** a Department of Epidemiology and Biostatistics, School of Public Health, MRC Centre for Environment and Health, Imperial College London, London, United Kingdom; b Division of Pulmonary, Critical Care, and Sleep Medicine, Department of Medicine, Beth Israel Deaconess Medical Center, Boston, Massachusetts; c PSE Healthy Energy, Oakland, California; d Division of Environmental Medicine, Depts. of Medicine and Population Health, New York University Grossman School of Medicine, New York City, New York; e Institute of Epidemiology and Medical Biometry, Ulm University, Germany; f ISGlobal, Barcelona, Spain; g Boston College, Newton, Massachusetts; h Division of Population Sciences, Dana-Farber Cancer Institute, Boston, Massachusetts; i Department of Environmental Health, Harvard T. H. Chan School of Public Health, Boston, Massachusetts; j Center for Health and the Global Environment, University of Washington, Seattle, Washington; k Department of Public Health, University of Copenhagen, Copenhagen, Denmark; l Centre for Environmental Policy, Imperial College London, London, United Kingdom; m School of Public Health, University of Haifa, Israel

The negative impacts of global climate change are well-known, but the health benefits of climate mitigation actions and their high monetary valuations are less appreciated. Actions to reduce the use of fossil fuels and greenhouse gas (GHG) emissions from energy production, industry, transportation, and agriculture will also bring major benefits to public health (see Figure 1).^1^ Economic benefits from health care savings and improved population health can far exceed the cost of climate mitigation measures. Cities, regions, and countries that implement climate change mitigation actions will gain immediate and long-term economic benefits of these health improvements.

**Figure 1.**
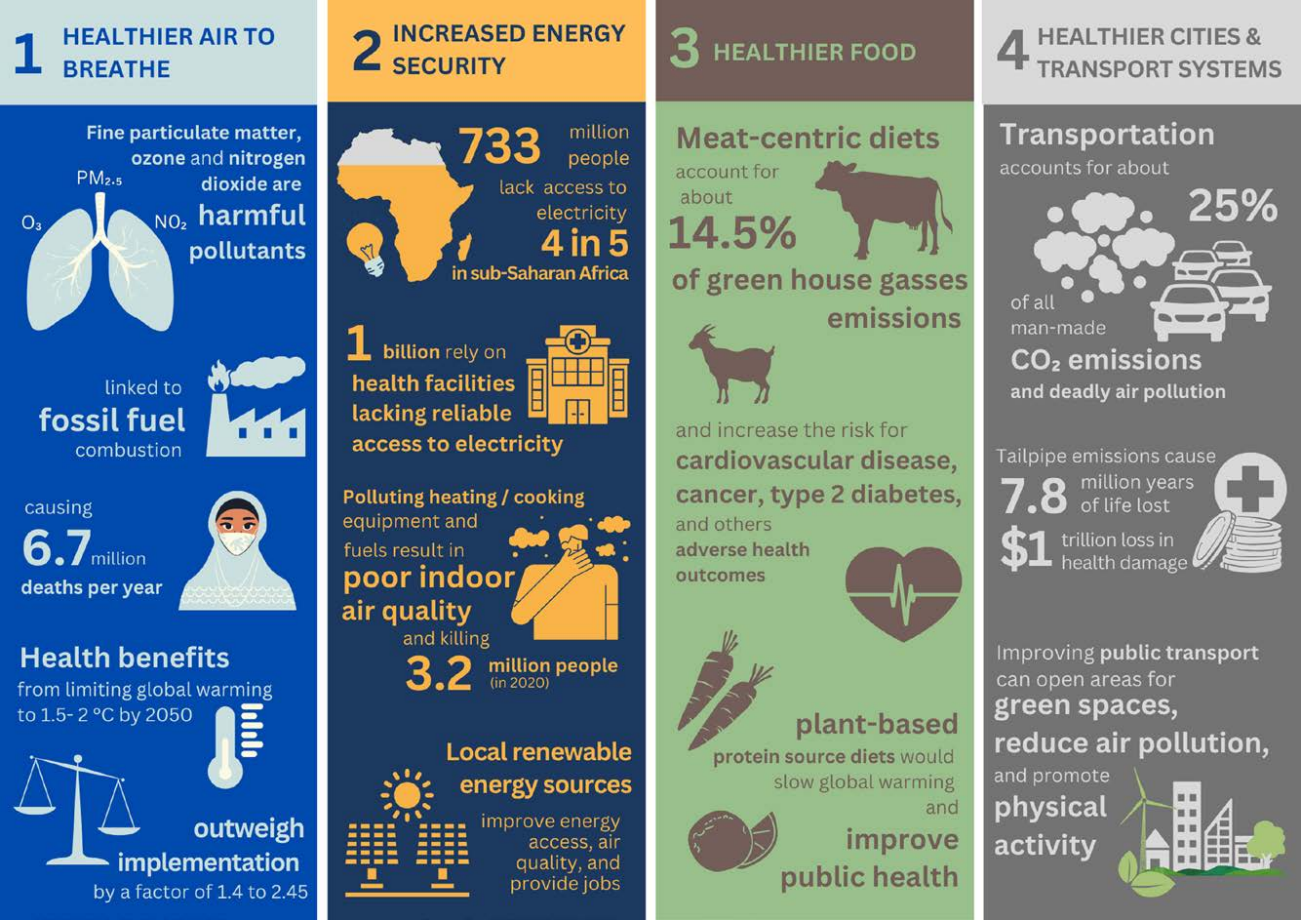
Health benefits of climate change actions.

## Climate action cobenefit 1: healthier air to breathe

Reducing the use of fossil fuels and transitioning to renewable energy improves air quality. Fine particulate matter, ozone, and nitrogen dioxide are harmful air pollutants originating from fossil fuel combustion. Air pollution is a major environmental hazard that contributes to 6.7 million premature deaths per year,^2^ and to multiple debilitating diseases such as cardiovascular diseases, chronic and infectious respiratory diseases, type 2 diabetes, lung cancer, and dementia. The value of health savings from improving air quality to limit global warming to 1.5–2 °C by 2050 outweighs the implementation costs by a factor of 1.4 to 2.45.^3^ The averted premature mortality from air pollution reductions will produce annual monetized benefits in the hundreds of billions of dollars over the next few decades, rising to several trillion annually at the end of the century.^4^ The air quality benefits of climate mitigation are immediate, and those living in regions that reduce fossil fuel combustion will experience not only immediate improvements in air quality but also a significant reduction in associated health risks.

## Climate action cobenefit 2: increased energy security

Switching to renewable energy sources can increase energy security. Energy access, health, and well-being are all inextricably linked. Energy is required for cooking, lighting, heating, cooling, housekeeping, and medical care-related activities, including life-sustaining medical devices. Nonetheless, 733 million people lack access to reliable electricity and cannot adequately meet their energy needs–3 in 4 are in Sub-Saharan Africa.^5^ Lack of access to electricity and high energy costs forces many households to rely on heating and cooking equipment and fuels that impair indoor air quality. Household air pollution has been linked to an increased risk of cardiovascular diseases, chronic obstructive pulmonary disease, and lung cancer, causing an estimated 3.2 million deaths annually.^6^ Additionally, about one billion people worldwide rely on health facilities that lack reliable access to electricity.^7^ Limited access to energy has a negative impact on the operational efficacy of healthcare facilities and the quality, accessibility, and dependability of services provided. Local renewable energy sources such as solar mini-grids^8^ improve energy access for households and healthcare facilities, provide jobs, improve air quality, and support the health of communities.

## Climate action cobenefit 3: healthier food

Changing food production to more climate-friendly foods provides significant human health benefits, especially by transitioning away from meat-centric diets. Livestock raised for animal-based food accounts for about 14.5% of global human-induced GHG emissions, mainly from methane, a potent GHG emitted by ruminant animals (cattle, sheep, and goats).^9^ Methane is also an ozone precursor. Ozone air pollution contributes to respiratory illnesses and loss of crops. For example, in 2015, estimated wheat production losses for Europe due to ozone pollution were 23.8 million tons, greater than Ukraine’s annual production.^10^ The transformation of land for livestock farming is causing a rapid decline in carbon sinks—the terrestrial biosphere absorbs 30% of anthropogenic CO_2_ emissions.^11^ Importantly, meat consumption contributes to poor health, including risk of cardiovascular disease, type 2 diabetes, and cancer.^12^ Plant-based sources of protein (e.g., legumes, peanuts, soybeans, chickpeas, and lentils) are healthier and contain less saturated fat.^13^ Transitioning meat production to plant-based proteins would rapidly slow global warming by cutting methane and would also improve health.

## Climate action cobenefit 4: healthier cities and transportation systems

Cities account for 70% of the global GHG emissions and are therefore a crucial target for climate change mitigation actions.^14^ Transportation accounts for nearly a quarter of all man-made CO_2_ emissions worldwide.^15^ Most (95%) of the energy used for transportation still comes from fossil fuels, representing 57% of global oil demand and 28% of total energy consumption.^15^ Traffic harms health in multiple ways.^16^ Emissions from trucks, cars and buses are among the largest sources of deadly air pollution in high- and low-income countries.^17^ Tailpipe emissions cause more than 7.8 million years of life lost and $1 trillion in health damages per year.^18^ Transportation noise is the second leading environmental stressor after air pollution, causing annoyance, sleep disturbance, and cardio-metabolic diseases.^19^ Additionally, motorized commutes are a missed opportunity for commuters to engage in physical activity.^14^ More than a quarter of the world population is insufficiently active, contributing to more than 7% of premature deaths globally and accounting for more than $50 billion in health care costs.^20^ While current transportation schemes generate air pollution, noise, and heat and contribute to the urban heat island in cities,^21^ green space attenuates these harmful exposures and can remove CO_2_ from the atmosphere. When space currently occupied by cars is repurposed for public green spaces, it can promote outdoor physical activity, improve air quality, reduce noise and enhance mental well-being and social cohesion.^14^ Climate change action can promote health through a redesign of cities and transport networks, encouraging shifts away from polluting vehicles and traffic, and improving accessibility to goods and services (including healthcare) in cities, while dedicating more space to health-enhancing land uses such as greenspace, walking and cycling.

### Take-away message

Climate action will improve health by providing cleaner air, energy security, healthier foods, sustainable and active transportation, and building more walkable, inclusive and livable cities and communities. Applying an equity lens in all these policies is essential to ensure equitable access and distribution of the many health benefits of climate action. Climate change mitigation policies must consider the health benefits of action.

*This commentary was written by the Policy Committee of the International Society for Environmental Epidemiology (ISEE). We thank all the committee members for their valuable comments.*
